# Germline mutation within *COL2A1* associated with lethal chondrodysplasia in a polled Holstein family

**DOI:** 10.1186/s12864-017-4153-0

**Published:** 2017-10-10

**Authors:** Sina Reinartz, Hartmut Mohwinkel, Christian Sürie, Maren Hellige, Karsten Feige, Deborah Eikelberg, Andreas Beineke, Julia Metzger, Ottmar Distl

**Affiliations:** 1 Institute for Animal Breeding and Genetics, University of Veterinary Medicine Hannover, Hannover, Germany; 2 Teaching and Experimental Farm Ruthe, University of Veterinary Medicine Hannover, Ruthe, Hannover, Germany; 3 Clinic for Horses, University of Veterinary Medicine Hannover, Hannover, Germany; 4 Institute for Pathology, University of Veterinary Medicine Hannover, Hannover, Germany

**Keywords:** Cattle, Whole genome sequencing, Bulldog, *COL2A1*, Mosaic

## Abstract

**Background:**

The bulldog calf syndrome is a lethal form of the inherited congenital chondrodysplasias. Among the progeny of the polled Holstein bull Energy P cases of lethal chondrodysplasia were observed. Pedigrees of the cases and the frequency of 3/8 cases among the offspring of Energy P at our teaching and experimental farm Ruthe (LuFG Ruthe) supported the assumption of a germline mutation with a mosaic of normal and defective sperm.

**Results:**

All three malformed calves were examined using necropsy, histopathology and computed tomography scanning. The phenotypic appearance of the affected calves was highly similar; they presented with severe disproportionate dwarfism and reduced body weight. The syndrome was characterized by brachygnathia superior, bilateral palatoschisis, shortening and compression of the body due to malformed vertebrae, in their size reduced and malformed ribs and reduced length of the long bones of the limbs. The bones had small irregular diaphyses and enlarged epiphyses. Whole genome sequencing of one bulldog calf, sperm of its sire Energy P and a normal progeny of Energy P identified a deleterious missense mutation (g.32476082G > A, c.2986G > A, ss2019324576) within *COL2A1* on bovine chromosome (BTA) 5. Sanger sequencing confirmed the ss2019324576 variant in the affected calves and sperm of Energy P. This mutation is located within the collagen triple helix repeat and causes an exchange of glycine to serine (p.996G > S) in COL2A1. This private single nucleotide variant (SNV) was present as a gonadal mosaic in sperm of the bull. All affected calves were in a heterozygous state whereas normal half-siblings and all dams of the progeny from Energy P were missing this SNV. Validation in polled Holstein bulls and normal Holstein calves randomly sampled from several herds and from the LuFG Ruthe confirmed this SNV as private.

**Conclusions:**

The identified spontaneous missense mutation within *COL2A1* is most likely the cause of lethal chondrodysplasia in the progeny of Energy P through a dominant negative effect. This example suggests that it would be beneficial to conduct whole genome sequencing of sperm from bulls widely used in artificial insemination in order to detect germline mosaicism.

**Electronic supplementary material:**

The online version of this article (10.1186/s12864-017-4153-0) contains supplementary material, which is available to authorized users.

## Background

The “bulldog calf” is a term used for various forms of inherited congenital chondrodysplasias in cattle. In Dexter cattle the disease is associated with two mutations (BD-1 and BD-2) in the *aggrecan* gene (OMIA 001271–9913, dwarfism, aggrecan type in *Bos taurus* (cattle), gene: *ACAN,* http://omia.angis.org.au/) [1]. In miniature Highland cattle, a case of chondrodystrophic dwarfism with hydrocephalus due to the BD-1 mutation in *ACAN* was demonstrated [2]. In several cattle breeds from Europe and USA, “bulldog calf” phenotypes have been reported [2–6]. These cases are characterized by a disproportionate dwarfism showing severely shortened limbs, a short and compressed body, facial dysplasia and additional malformations (OMIA 001926–9913 achondrogenesis, type II in *Bos taurus* (cattle), gene: *COL2A1*, http://omia.angis.org.au/). The French Holstein bull “Igale Masc”, widely used in artificial insemination, sired a small proportion (~1%, *n* = 75) of his calves with the congenital chondrodysplasia of the “bulldog calf” phenotype [7]. All sampled 75 affected calves did not show runs of homozygosity and thus, recessive inheritance seemed very unlikely. In addition, repeated matings among “Igale Masc” and 32 dams which gave birth to a “bulldog calf” in the previous calving could not produce the same chondrodysplastic phenotype. Therefore, a dominant mutation from a mosaic sire appeared very likely. Filtering whole genome sequences from the 1000 bull genome project including the Holstein bull “Igale Masc” and two affected calves for heterozygous mutations yielded two heterozygous mutations shared by the affected calves and “Igale Masc”. One of the two mutations was located within the candidate gene *COL2A1* (*COL2A1*:g.32475732G > A, c.2986G > A, p.Gly960Arg). Further genotyping of the g.32475742G > A mutation in affected calves and controls confirmed heterozygosity of this mutation and a perfect association with the “bulldog calf” phenotype. The most likely explanation was a mosaicism of the sire “Igale Masc” in his germ line. This missense mutation was predicted to disrupt the invariant Gly-X-Y structural motif of COL2A1 and reduced secretion of type II collagen.

Another case of a dominant mosaic mutation within *COL2A1* was confirmed for the Danish Holstein bull VH Cadiz Captivo [6]. A splice site mutation (c.2463 + 1G > A) was predicted to alter the splice donor sequence GT at the 5′-end of intron 36 and cause a change to AT. All five cases were heterozygous and the dams homozygous wildtype. Approximately 12% of the offspring were affected by lethal chondrodysplasia with the bulldog phenotype.

Mutations in *COL2A1* cause a wide spectrum of skeletal disorders in humans including achondrogenesis type II (ACG2) (OMIM 200610, achondrogenesis, type II; ACG2) [8, 9] which is characterized by short limbs, short ribs, and absence or abnormal ossification of some skeletal parts [10, 11].

In the Holstein cattle herd at the teaching and experimental farm Ruthe (LuFG Ruthe) three “bulldog calves” were delivered at term in 2016. All affected calves were non-viable and died during or shortly after birth. All affected calves were sired by the polled Holstein sire Energy P. This Holstein sire was heterozygous at the polled locus according to the official genotyping results of the artificial insemination (AI) station. “Bulldog calves” and similar chondrodysplastic malformations have not been observed in this herd as long as veterinary reports are available. The objective of this work was to elucidate the mode of inheritance and map this lethal malformation using the Illumina bovine high density beadchip with 777,962 SNPs (single nucleotide polymorphisms) on the bovine genome. Whole genome sequencing analysis of DNA was conducted with the aim to identify a deleterious and perfectly co-segregating mutation. In order to prove a germline mutation, we also sequenced DNA from semen samples of the bull, which sired the affected calves.

## Results

### Phenotype

**Fig. 1:**
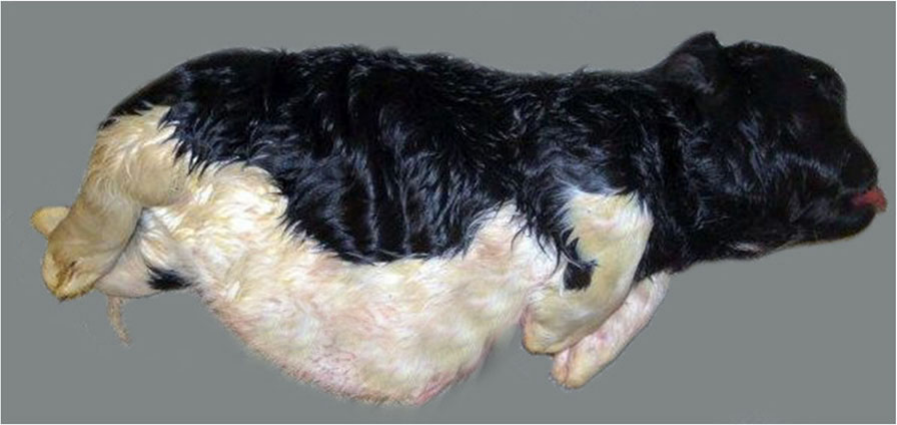
Lateral view of bulldog calf 2 delivered at term from a normal Holstein cow. The short and compressed body, bulky abdomen, symmetrically shortened limbs and brachycephalic face resemble the bulldog calf

**Fig. 2:**
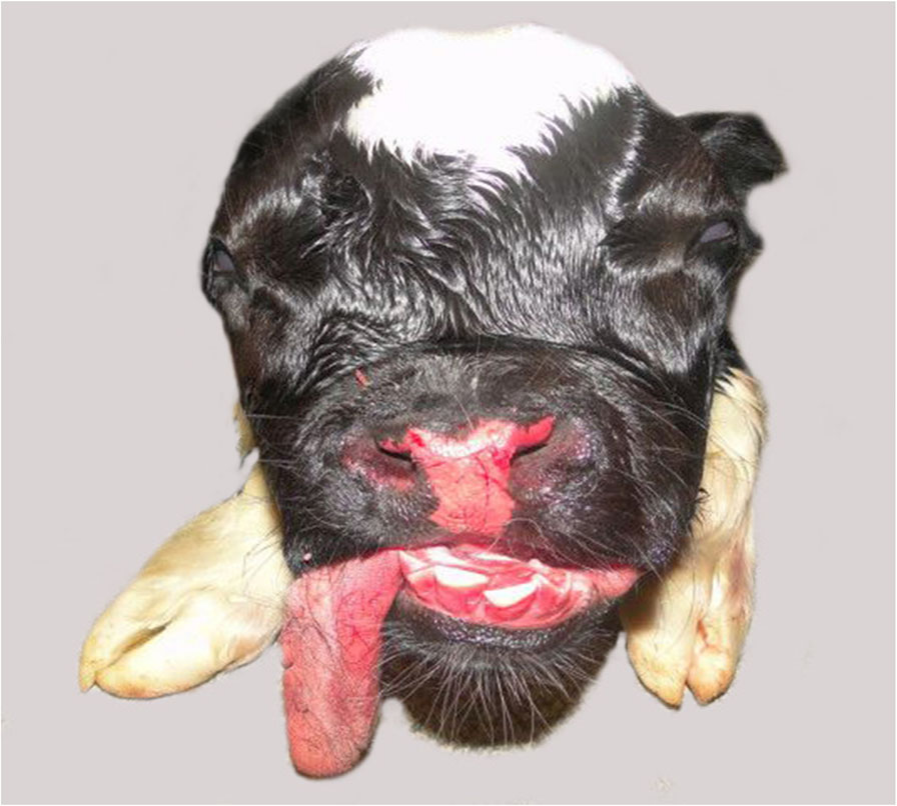
Frontal view of the face of bulldog calf 2. The frontal bossing, the caudal displacement of the eyes and the marked shortening of the upper jaw and protrusion of the tongue were striking features for the brachycephalic skull

On gross examination, the phenotypic appearance of the malformation of the three affected calves (bulldog calves 1–3) was highly similar. The calves were born at term and fully developed, but were dead at birth or died shortly after birth. Bulldog calf 1 was born after 251 days and weighed 16.3 kg. Bulldog calf 2 weighed 27.5 kg after 278 days of gestation and bulldog calf 3 weighed 18 kg after 273 days of gestation. The hair coats of the affected calves were fully developed. Wrinkles of the skin were not obvious. The short and compressed bodies with bulky abdomen, severe bilateral symmetrical shortening of the limbs and severe facial dysmorphisms resembled the “bulldog calf” phenotype with an extremely disproportionate chondrodysplasia (Fig. 1). The faces were characterized by frontal bossing and marked shortening of the muzzles resulting in a protrusion of the tongues (Fig. 2). Bilateral palatoschisis besides the midline with an opening of the hard and soft palate was evident in all three cases. The limbs were severely reduced in length but had normally sized digits.

**Fig. 3:**
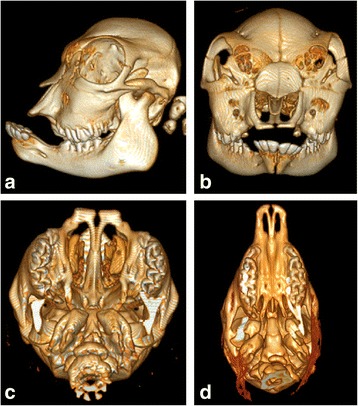
Computed tomography of the skull of the bulldog calf 2 (**a**-**c**) and a control animal (**d**). The control animal was a Vorderwald fetus which was stillborn after 8 months of gestation. **a** Left lateral view displays brachygnathia superior with prolonged lower jaws (prognathism). The splanchnocranium was dysplastic in opposite to the prominent neurocranium. **b** In the rostrocaudal view the shortening of the nasal bones, bossing of the os frontale and the caudodorsal displacement of the orbitae are evident. **c** View of the oral cavity roof (mandibles had been removed). The compressed, spherical shape of the skull and the bilateral cleft palate were striking features. **d** The skull of the Vorderwald fetus shows an elongated and slender shape without cleft palate

**Fig. 4:**
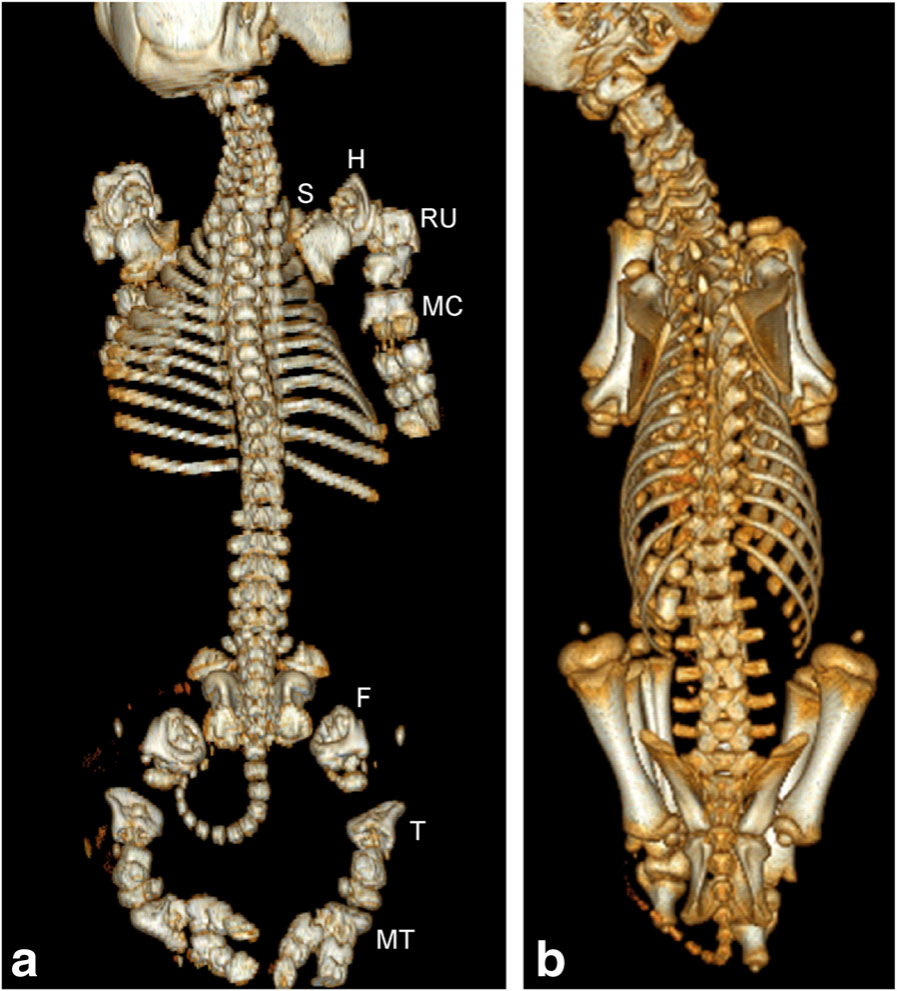
Computed tomography scans of the bulldog calf 2 (**a**) and the control animal (**b**). The bones appearing chondrodysplastic are indicated by letters: S, scapula, H, humerus, RU, radius/ulna, MC, metacarpus, F, femur, T, tibia, MT, metatarsus. The dorsal view of both animals enables comparative analysis of both skeletal systems. The ribs, the lumbar spine, the pelvis and the skeleton of the limbs seem particularly malformed in the bulldog calf. The ribs are malformed and short. The opening angle is larger than in the normal calf. Especially the vertebrae of the lumbar and cervical spine appeared flat (platyspondylia) and the processus transversi of the lumbar spine are completely missing. The pelvis was significantly retarded in its development. The skeleton of the limbs were severely malformed. Especially the long bones above the phalanges were short and irregularly shaped

Computed tomography (CT) was done for all three bulldog calves. The morphology of the skeletons of the three cases was very similar to each other. A severe shortening of the nasal and the incisive bones was evident. The calves had brachygnathia superior with a prolonged lower jaw (prognathism). The splanchnocrania were dysplastic with caudodorsal displacement of the orbitae in opposite to the prominent neurocrania (Fig. 3a, b). These altered ratios provided a spherical appearance of the skull, which is particularly evident in comparison with a non-affected animal (Fig. 3c, d). The chondrodysplastic animals also had a bilateral cleft palate extending through the hard and soft palate. In the comparative examination of the body skeleton of bulldog calf 2 with a phenotypically normal calf, particularly the ribs, the lumbar spine, the pelvis and the skeleton of the limbs had striking malformations (Fig. 4). The ribs were short and distances among them were irregular but the numbers were normal. The opening angle was larger than in the normal calf. The ribs extended more horizontally instead of vertically. All vertebrae were developed and their number did not deviate from normal calves. Especially the vertebrae of the lumbar spine were severely malformed. They were flat (platyspondylia) and the processus transversi of the lumbar spine were completely missing. The vertebrae of the cervical spine were flatter than in the control animal. The pelvis was severely retarded in its development. Its shape was small and stocky. The long bones of the limbs above the phalanges were short and irregularly shaped. The bones of the limbs were poorly mineralized and consisted mainly of cartilage.

### Gross and microscopic findings

**Fig. 5:**
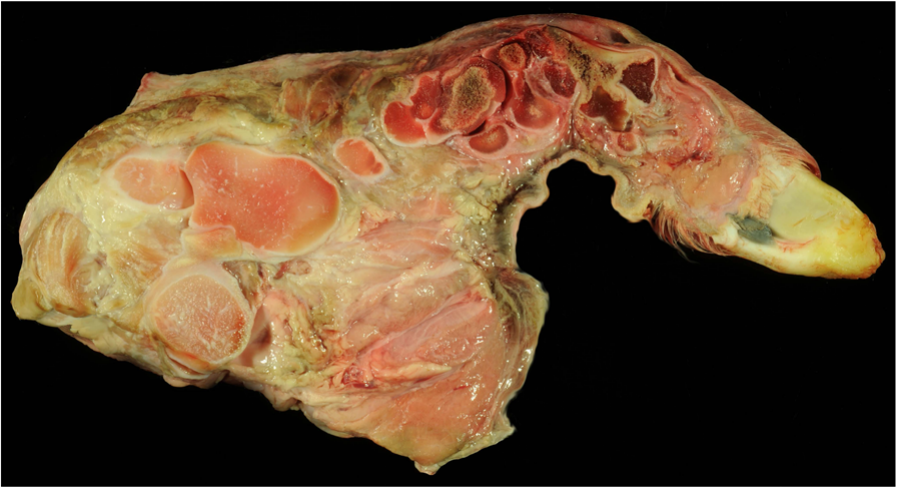
Malformed limb with shortened and irregular formed long bones from bulldog calf 2

**Fig. 6:**
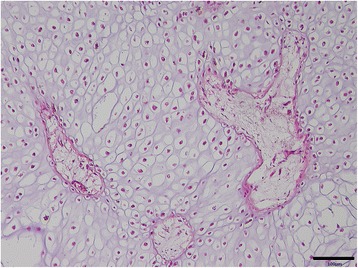
Long bone with irregular arranged hypertrophic chondrocytes in the metaphyseal region of bulldog calf 2. Hematoxylin eosin stain, bar = 100 μm

**Fig. 7:**
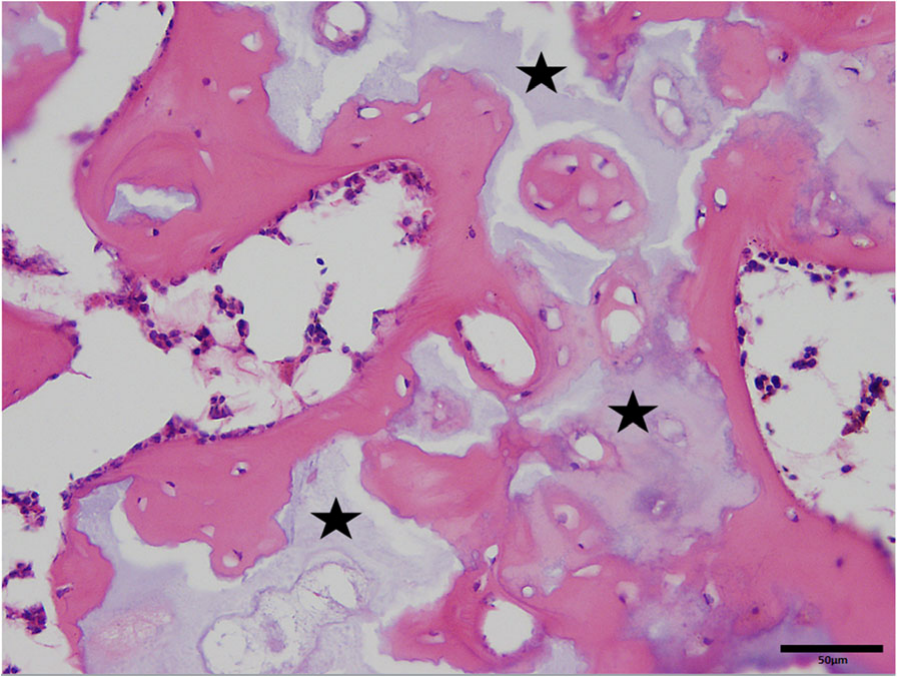
Vertebral bone with islands of retained cartilage (marked with asterisks) of bulldog calf 2. Hematoxylin eosin stain, bar = 50 μm

Post mortem examination demonstrated very similar findings in all three affected calves as exemplified for bulldog calf 2. A diffuse subcutaneous edema (anasarca) was seen in the three present cases. Skeletal abnormalities were characterized by markedly shortened and partially rotated limbs (Fig. 5) and reduced length of the vertebral column. The thorax was dorsoventrally flattened. The head was dome shaped with a protruded mandible and cleft palate. Histology of malformed limbs showed irregular proliferations of densely packed and hypertrophic chondrocytes without distinct growth plates (Fig. 6), indicative of disturbed enchondral ossification. Islands of retained cartilage with thin osteoid layers were found in the epiphysis, metaphysis and diaphysis (Fig. 7) of malformed bones. In addition, articular cartilage was thickened and showed irregular proliferations and disorganization of chondrocytes.

### Pedigree analysis

Screening of the veterinary records of the herd of the LuFG Ruthe gave no indication for the occurrence of a similar malformation in the past 40 years. All newborn calves and all aborted calves are under veterinary control and any congenital abnormalities are recorded in a computerized herd management program. The dams, their progeny and any ancestors which were still at LuFG Ruthe were examined but no indications for skeletal abnormalities were found.

**Fig. 8:**
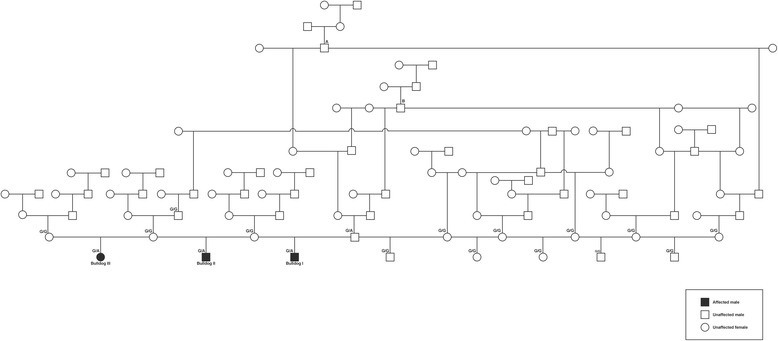
Pedigree of the three bulldog calves sired by the polled Holstein bull Energy P. The polled Holstein bull Energy P has a normal phenotype and three progeny show the bulldog form of chondrodysplasia. Sires A and B are common ancestors of Energy P and dams sired by Energy P. Energy P is inbred on sire B. A normal progeny from Energy P is inbred on ancestor B and another normal progeny from Energy P is inbred on ancestors A and B

The pedigree revealed that all bulldog calves were sired by one polled Holstein bull used for AI. All females inseminated with semen of Energy P got pregnant and no abortion was observed in these animals. In total, 8 calves were sired by this bull and 3/8 calves were bulldog calves (Fig. 8). Inbreeding in the affected calves on ancestors of Energy P could not be seen, whereas two dams of unaffected calves were related with Energy P via male ancestors (sires A and B in the pedigree). Energy P was inbred on a male ancestor (sire B in the pedigree).

### DNA analysis

We performed a runs of homozygosity (ROHs) in windows of 15 SNPs for the three bulldog calves using 761,461 SNPs from the Illumina bovine high density beadchip (Illumina, San Diego, CA) using PLINK, version 1.07 (http://pngu.mgh.harvard.edu/purcell/plink/). We were not able to identify consensus homozygosity regions for all three affected calves. The three bulldog calves had 356, 370 and 389 ROHs with an average size of 350, 458 and 344 kb, respectively. We rerun this analysis after having removed all SNPs not being in Hardy-Weinberg-equilibrium (*P* < 0.00000001). However, we were not able to find consensus ROHs for the three bulldog calves. Subsequently, we employed Illumina whole genome sequencing for one bulldog calf, sperm of its sire Energy P and a normal progeny of Energy P to search for variants shared by a male bulldog calf and the sperm sample of its sire but not shared by a normal male paternal half sibling of the bulldog calf. In addition, we used 19 bovine whole-genome sequences from four different breeds as controls. Reads were mapped to the UMD3.1 bovine reference genome resulting in a mean coverage of 12.4X for the bulldog calf, 13.2X for sperm of Energy P, 8.6X for the normal progeny of Energy P and 10-15X for the 19 controls. Raw data including the Energy P family and the 19 controls contained 29,848,582 variants whereof 2,039,166 were unknown in dbSNP (https://www.ncbi.nlm.nih.gov/snp/).

Filtering for variants with high, moderate or low effects according to SNPEff predictions on the protein structure and variants associated with chondrodysplasia-related genes left only one variant within *COL2A1* on BTA5 (UMD3.1). We were not able to identify mutations predicted to have a moderate or high-impact effect on protein structure in any other chondrodysplasia-related gene (Additional file 1). The variant within the bovine gene *COL2A1* was a heterozygous missense mutation (g.32476082G > A, c.2986G > A, ss2019324576) within exon 42 based on the transcript variant *COL2A1–201* (Ensembl). This mutation causes an exchange of glycine to serine (p.996G > S). Using SIFT [12] and PolyPhen-2 [13] this missense mutation was predicted to be deleterious with a score of 0 (SIFT) or damaging with a score of 1.0 (PolyPhen-2). A search of the NCBI and Ensembl databases for SNPs could not retrieve this missense mutation.

**Fig. 9:**
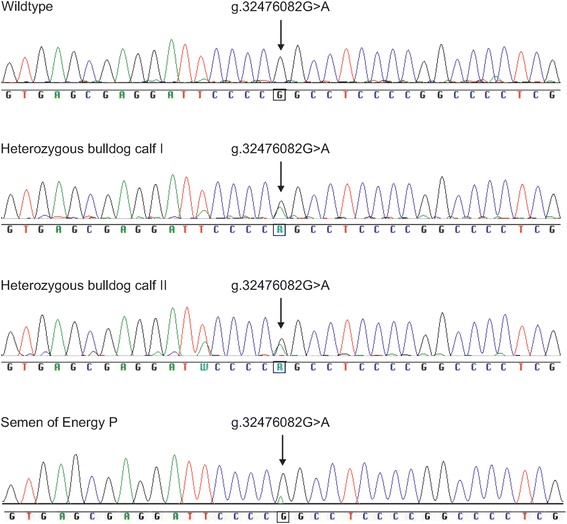
Mutation analysis of exon 42 of *COL2A1* in two bulldog calves, a control animal and a semen sample of Energy P. Shown is the heterozygous missense mutation g.32476082G > A for two bulldog calves and the homozygous wildtype of the control animal. The semen sample of Energy P revealed the presence of the mutant allele A with a small sized peak

Sanger sequence analysis of the *COL2A1* gene for two bulldog calves and an unrelated Holstein Friesian control animal from another herd as shown in Fig. 9 confirmed the ss2019324576 variant in bulldog calves and showed the absence in the control. The semen sample of Energy P revealed the presence of the mutant allele A with a much smaller size peak than the samples from the bulldog calves. The estimated ratio among the area under allele A in comparison to the area of both alleles was approximately 5%. We developed a PCR (mismatch)-restriction fragment length polymorphism (PCR-RFLP, Additional file 2) and validated this PCR-RFLP in the three bulldog calves and the semen sample of Energy P (Additional file 3). The dams of the three bulldog calves, an unrelated control animal (Additional file 3) and all five normal progeny of Energy P were homozygous wildtype (Additional file 4).

Subsequently, we genotyped 105 polled Holstein bulls and 105 normal Holstein calves whereof 48 were descendants from cows of the dairy herd of the LuFG Ruthe and in addition, 66 animals from other cattle breeds using the PCR-RFLP for the ss2019324576 variant (Additional file 2). We were not able to identify this mutated allele in any other individuals (Additional file 4). Therefore, we performed a genome-wide association study (GWAS) for the three affected calves, 96 polled Holstein bulls and 96 normal Holstein calves using the SNPs of the Illumina bovine high density beadchip and the ss2019324576 variant. After quality control and exclusion of beadchip SNPs with a minor allele frequency (MAF) <0.05, 586,979 SNPs were left for the GWAS. The GWAS gave a highly significant peak for the ss2019324576 variant (*P*-value = 6.436e-30, after Bonferroni correction for multiple testing), whereas all other SNPs were not significant (*P*-value > 0.05, after Bonferroni correction for multiple testing). These GWAS results support that this *COL2A1*-associated mutation is very likely a spontaneous germline mutation and chondrodysplasia causing mutations in other genomic and non-coding regions are unlikely.

We calculated *COL2A1* containing haplotypes for all 195 animals using beadchip SNPs and the ss2019324576 variant (SAS/Genetics, Statistical Analysis System, Cary, NC). These haplotypes were restricted to 15 SNPs spanning 46,558 bp from 32,441,205 to 32,487,763 bp on BTA5. We searched for the allelic pattern of the haplotype surrounding the ss2019324576 variant and associated with the mutant allele A in affected calves. After having identified this haplotype only shared by the three bulldog calves (A-A-C-G-A-A-A-G-A-G-[**A]**-G-G-G-G) we searched all normal animals for the proximally and distally of the ss2019324576 variant identical haplotype (A-A-C-G-A-A-A-G-A-G-[**G]**-G-G-G-G). In 14.5% of the normal animals, this latter haplotype was present. This might indicate that the haplotype with the wildtype allele G of the ss2019324576 variant may be common among Holsteins, particularly in polled Holstein sires and furthermore, the three bulldog calves share the ss2019324576 A-variant surrounding haplotype with some of the normal calves.

## Discussion

The present study demonstrates the power of whole genome sequencing to detect a lethal dominant germline mutation using a sample from the sperm of the sire, one affected and one normal half-sibling. Filtering for deleterious variants only shared between the affected calf and the sire gave just one hit in the candidate gene *COL2A1.* Validation in immediate and more distant relatives confirmed the absence of the *COL2A1* mutation (ss2019324576). In addition, there were no indications of shared genomic regions or associated regions among affected calves. The ss2019324576 variant surrounding haplotype but without the mutant allele A may be common in normal Holsteins and may support the assumption of a *de-novo* origin of the ss2019324576 variant. Based on this data, a germline mosaicism is very likely for the sire Energy P.

Heterozygous dominant mutations of *COL2A1* associated with bulldog calves have been identified in progeny of a French [7] and a Danish Holstein bull [6]. The gross, microscopic and CT findings were highly similar with these previous cases in Holsteins [3, 6, 7] as well as in Dexter [1] and miniature Highland cattle [2]. The lesions found in bulldog calves are consistent with the signs of a generalized chondrodysplasia [1–7].

The rare autosomal dominant type II collagenopathies with spondyloepiphyseal dysplasia are in humans accompanied by ocular and otolaryngological abnormalities [10, 11, 14, 15]. Several human *COL2A1* defects are dominant-negative mutations disrupting the triple-helical region of alpha 1 (II) chains with more severe phenotypes like achondrogenesis type II and hypochondrogenesis. These phenotypes are similar to the bovine *COL2A1*-associated chondrodysplasia in Holstein cattle [7]. Ocular and otolaryngological abnormalities were not shown in the present cases nor reported in cattle with bovine *COL2A1*-associated chondrodysplasia [1–7]. The p.996G > S missense mutation is located in the collagen triple helix repeat of *COL2A1* and may be assumed to disrupt the triple-helical region of alpha 1 (II) chains and cause a dominant-negative effect. Many of the most damaging mutations to collagen genes result in the substitution of a glycine residue within the triple helix. The repeating Gly-X-Y structure (where X and Y can be any amino acid) is mandatory for a tight packing of the polyproline II-type helices within the triple helix [16].

The frequency of bulldog calves among approximately 120 Energy P progeny was estimated at approximately 20% based on records of the German AI organization Masterrind, Verden/Aller, Germany, where Energy P was employed for AI [17]. This frequency depends on the proportion of germ cells carrying this *COL2A1* mutation. The high frequency may indicate that this spontaneous mutation occurred early in the embryonic development resulting in this high frequency of mutated sperm cells. An early reporting system and genetic analysis of sires before entering AI would prevent the high risk of allowing a dominant paternal mutation from entering the broader animal population. This example may also show that whole genome analysis of sperm of bulls widely used in artificial insemination (AI) should be performed to avoid lethal malformations due to spontaneous dominant mutations in the first generation of progeny and dissemination of recessive mutations in the first and later generations.

## Conclusions

The present study suggested that the strongly with lethal chondrodysplasia syndrome associated ss2019324576 variant in a polled Holstein bull family may be a germline mutation. This mutation is likely to have disrupted triple-helical region of alpha 1 (II) chains of COL2A1 and exerting a dominant negative effect on the COL2A1 protein action. Paternal *de-novo* dominant and recessive mutations may be very early detected by employing whole genome sequencing data analysis of sperm from AI-bulls.

## Methods

### Animals

Genomic DNA was obtained from blood and tissue samples of five normal and three affected progeny of the Holstein bull Energy P, their Holstein dams and sperm from Energy P. In addition, 48 EDTA blood samples from Holstein calves born on the LuFG Ruthe, further 57 randomly collected Holstein calves and 105 polled Holstein bulls were available. Furthermore, 66 blood samples from five different cattle breeds and a crossbred were employed for the present study.

### Computed tomography

The CT image acquisition was performed in sternal recumbency of the affected calves. CT scans were acquired with a multislice helical CT scanner (Brilliance-TM-CT-16 BigBore, Philips Healthcare, The Netherlands) using conventional settings (90 kV, 100 mAs) and 2 mm slice thickness.

### Histopathological examination

Bone and articular tissues were prepared for the affected calves with a diamond saw and formalin fixed for 48 h. Prior to paraffin embedding samples were decalcified in HNO_3_ solution (5%) for 24 h. Subsequently, 3 μm-thick sections were cut on a microtome, mounted on glass slides and stained with hematoxylin and eosin.

### Genotyping

Genomic DNA was extracted from the EDTA-blood samples using routine procedures. Concentration of extracted DNA was determined using the Nanodrop ND-1000 (Peqlab Biotechnology, Erlangen, Germany). DNA concentration was adjusted to 50–80 ng/μl. We performed a high-density genome-wide association analysis employing the Illumina bovine high density beadchip comprising 777,962 SNPs with a mean spacing of 3.4 kb across the entire bovine genome. Raw data were analysed using the genotyping module version 1.9.4 of the GenomeStudio V2011.1 software (Illumina). All SNPs underwent quality control. SNPs with a call rate < 0.95 were excluded from further analyses. Due to these specifications, 761,461 SNPs were left for runs of homozygosity (ROHs). Calculation of ROHs was done using PLINK, version 1.07 (http://pngu.mgh.harvard.edu/purcell/plink/). After exclusion of SNPs with a minor allele frequency (MAF) <0.05, 586,979 SNPs were left for the GWAS. In addition to this set of beadchip SNPs, the *COL2A1*:g.32476082G > A SNV was added for the analysis. The positions of all SNPs and the SNV were according to the UMD 3.1 bovine genome assembly.

### Whole-genome sequencing

DNA samples from one male bulldog calf, sperm of the bull Energy P and a normal male offspring of Energy P were prepared for sequencing using the NEBNext Ultra II DNA Library Prep Kit for Illumina for high quality libraries (NEB, New England BioLabs, Ipswich, MA) according to standard NEB-protocols. Size selection and indexing was quality controlled on an Agilent 2100 Bioanalyzer system with a High Sensitivity DNA kit (Agilent Technologies, Santa Clara, CA). Finally, indexed libraries were sequenced on the Illumina NextSeq 500 for 300 cycles in 2 × 150 paired-end modes. After quality control of fastq files using fastqc 0.11.3 (http://www.bioinformatics.babraham.ac.uk/projects/fastqc/) reads were mapped to the UMD3.1 bovine reference genome using BWA 0.7.12 [18], converted into binary format [19] and underwent variant calling using GATK [20]. Further variant effect prediction and classification of effects was done using SNPEff version 4.1 b [21, 22]. In addition, variant calling and bioinformatic analysis included 19 whole-genome sequences of different cattle breeds (Holstein, Galloway, Limousin, Vorderwald cattle) as controls. All fastq data can be obtained through the NCBI Sequence Read Archive (http://www.ncbi.nlm.nih.gov/sra), BioProject PRJNA350593 (BioProject submission: SUB2036163, SRA experiment IDs: SRX2268677, SRX2268678, SRX2268680).

### Variant filtering

Variants from whole-genome sequencing analysis were filtered for high, moderate or low effects according to SNPEff predictions using SAS, version 9.4 (Statistical Analysis System, Cary, NC). Private variants exclusively found in the bulldog calf and Energy P, but not in the normal male half-sib of the bulldog calf and further reference cattle data were filtered out and further controlled for dbSNP entries. Predicted effects of these private variants were investigated using the Variant Effect Predictor [22] for SIFT [12] predictions and PolyPhen-2 [13]. All variants heterozygous for the mutant allele in the bulldog calf and Energy P, but homozygous wildtype in the normal male half-sib as well the control animals were evaluated for their potential impact on chondrodysplasia using a candidate gene list [6] and GO terms. All candidate genes with potential protein structure influencing mutations were visually searched for mutations and deletions >10–15 bp using the Integrative Genomics Viewer (IGV, 2.3.x, http://software.broadinstitute.org/software/igv/) [23, 24].

### Sanger sequencing of *COL2A1*

Primer pairs were designed to amplify exon 42 of *COL2A1* (Additional file 5). These primer pairs had product sizes of 560 bp, 785 bp and 793 bp and included the respective exon with their adjacent introns. PCR was performed in 22-μl reaction volumes containing 2 μl genomic DNA, 70 μM deoxyribonucleoside triphosphates, 5 pmol of each primer, and 3.5 U of *Taq* polymerase in the reaction buffer supplied by the manufacturer (Qiagen, Hilden, Germany). After a 5-min initial denaturation at 94 °C, 36 cycles of 30 s at 94 °C, 1 min at 60 °C, and 45 s at 72 °C were performed in a T Professional Thermocycler (Whatman Biometra, Goettingen, Germany). The amplicons of bulldog calves 1–3, a semen sample of Energy P, three normal progeny of the sire Energy P and a further unrelated control animal were sequenced with forward and reverse sequencing primers on a Genetic Analyzer 3500 (Applied Biosystems by Life Technologies, Darmstadt, Germany). Sequences were analysed with Sequencher 4.8 (Genes Codes, Ann Arbor, MI, USA). Sequences were screened for mutations using the bovine reference sequence from NCBI (Bos_taurus_UMD_3.1.1, GCF_000003055.6) and Ensembl (UMD3.1, GCA_000003055.3).

### Validation

A PCR (mismatch)-restriction fragment length polymorphism (PCR-RFLP) was developed for the SNP g.32476082G > R. Primer pairs, amplicon size in base pairs (bp), annealing temperature, restriction enzyme and incubation temperature are given in Additional file 2.

## Additional files

Additional file 1: Results of the filtering analysis for variants heterozygous or homozygous mutant in a bulldog calf and a sperm sample of the sire Energy P but missing in an unaffected progeny of Energy P. Shown are the filtered genes containing variants with high- or moderate-impact on protein structure with their position on the bovine genome assembly UMD3.1 and a list of candidate genes for chondrodysplasia [6]. (XLSX 23 kb)

Additional file 2: Primer sequences used for validation of the mutation detected by Sanger sequencing. The heterozygous missense variant g.32476082G > R was validated using a restriction fragment length polymorphism (RFLP). Primer pairs, amplicon size (AS) in base pairs (bp), annealing temperature (AT), restriction enzyme and incubation temperature (IT) are given. Fragments in the wildtype exhibit sizes of 394 bp, 51 bp and 28 bp, whereas in the heterozygous animals, fragment sizes of 394 bp, 79 bp, 51 bp and 28 bp are present. (DOCX 14 kb)

Additional file 3: Genotyping of all three bulldog calves, a sperm sample of their sire Energy P, the dams of the bulldog calves and an unrelated control animal using a PCR restriction fragment polymorphism (PCR-RFLP). Samples with fragment sizes of 394 bp, 79 bp, 51 bp and 28 bp represent the heterozygous missense variant g.32476082G > R. (TIFF 538 kb)

Additional file 4: Animals genotyped for validation of the *COL2A1*:g.32476082G > A mutation in this study. Distribution of the g.32476082G > A genotypes in the family of Energy P (normal descendants, their dams and Energy P) in the herd where the bulldog cases were observed and 228 animals from different farms and breeds. Information about the polled genotype is included. Genotyping of the polled condition was according to Glatzer et al. (2013) [25]. (DOCX 17 kb)

Additional file 5: Primer pairs used for sequencing exon 42 of *COL2A1* on BTA5. Primer pairs, amplicon size (AS) in base pairs (bp) and the annealing temperature (AT) are given. (DOCX 14 kb)

## Abbreviations

*ACAN*: *aggrecan*
ACG2: achondrogenesis, type II
AI: Artificial insemination
BTA: Bovine chromosome
*COL2A1*: *collagen type II alpha 1 chain*
CT: Computed tomography
GWAS: Genome-wide association study
LuFG Ruthe: Teaching and experimental farm Ruthe
MAF: Minor allele frequency
NGS: Next generation sequencing
PCR-RFLP: PCR-restriction fragment length polymorphism
ROH: Runs of homozygosity
SNP: Single nucleotide polymorphism
SNV: Single nucleotide variant
