# Supplementary material for: Germline mutation within COL2A1 associated with lethal chondrodysplasia in a polled Holstein family
Source: BMC Genomics. 2017 Oct 10;18:762. doi: 10.1186/s12864-017-4153-0 (PMC5633883; doi:10.1186/s12864-017-4153-0)
Supplement: Supplementary file 2 — Primer sequences used for validation of the mutation detected by Sanger sequencing. The heterozygous missense variant g.32476082G > R was validated using a restriction fragment length polymorphism (RFLP). Primer pairs, amplicon size (AS) in base pairs (bp), annealing temperature (AT), restriction enzyme and incubation temperature (IT) are given. Fragments in the wildtype exhibit sizes of 394 bp, 51 bp and 28 bp, whereas in the heterozygous animals, fragment sizes of 394 bp, 79 bp, 51 bp and 28 bp are present. (DOCX 14 kb) [file 12864_2017_4153_MOESM2_ESM.docx]

**Additional file 2. Primer sequences used for validation of the mutation detected by sanger-sequencing.** The heterozygous missense variant g.32476082G>R was validated using a restriction fragment length polymorphism (RFLP). Primer pairs, amplicon size (AS) in base pairs (bp), annealing temperature (AT), restriction enzyme and incubation temperature (IT) are given. Fragments in the wildtype exhibit sizes of 394 bp and 79 bp, whereas in the heterozygous fragments with sizes of 394 bp, 79 bp, 51 bp and 28 bp are present.

| Gene | Genetic variant | Forward primer (5’-3’) | Reverse primer (5’-3’) | AS (bp) | AT (°C) | Restriction enzyme | IT  (°C) |
| --- | --- | --- | --- | --- | --- | --- | --- |
| *COL2A1-201* | g.32476082G>R | CCTGGACAGCGTGGTGAGCGAGGATTCGCC | TAGTCTCCCTGGCCTTCTCTC | 473 | 60 | NgoMIV | 37 |
