# Supplementary figures and images for: Germline mutation within COL2A1 associated with lethal chondrodysplasia in a polled Holstein family
Source: BMC Genomics. 2017 Oct 10;18:762. doi: 10.1186/s12864-017-4153-0 (PMC5633883; doi:10.1186/s12864-017-4153-0)

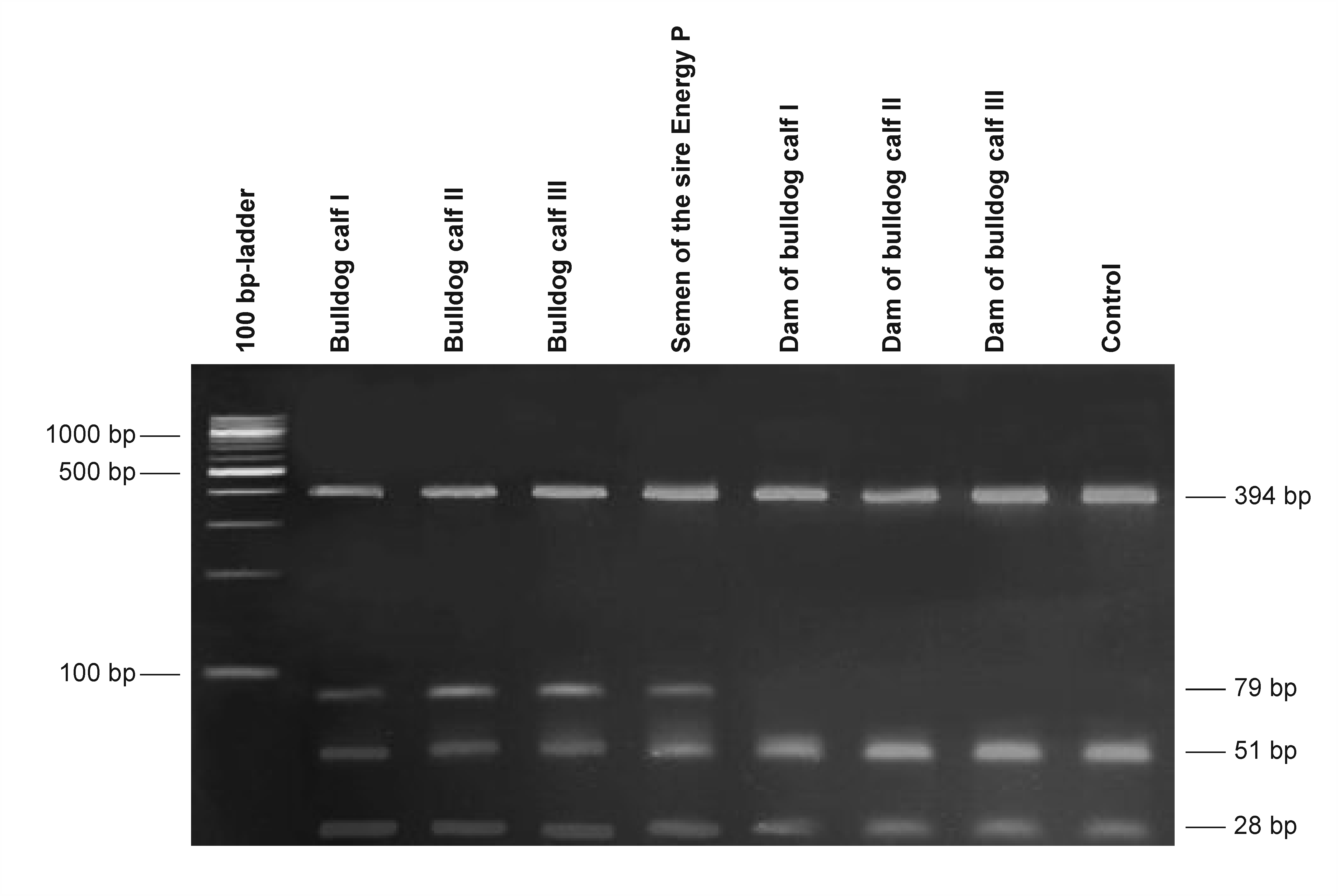

Supplement: Supplementary file 3 — Genotyping of all three bulldog calves, a sperm sample of their sire Energy P, the dams of the bulldog calves and an unrelated control animal using a PCR restriction fragment polymorphism (PCR-RFLP). Samples with fragment sizes of 394 bp, 79 bp, 51 bp and 28 bp represent the heterozygous missense variant g.32476082G > R. (TIFF 538 kb) [file 12864_2017_4153_MOESM3_ESM.tif]
