# Supplementary material for: Germline mutation within COL2A1 associated with lethal chondrodysplasia in a polled Holstein family
Source: BMC Genomics. 2017 Oct 10;18:762. doi: 10.1186/s12864-017-4153-0 (PMC5633883; doi:10.1186/s12864-017-4153-0)
Supplement: Supplementary file 4 — Animals genotyped for validation of the COL2A1:g.32476082G > A mutation in this study. Distribution of the g.32476082G > A genotypes in the family of Energy P (normal descendants, their dams and Energy P) in the herd where the bulldog cases were observed and 228 animals from different farms and breeds. Information about the polled genotype is included. Genotyping of the polled condition was according to Glatzer et al. (2013) [25]. (DOCX 17 kb) [file 12864_2017_4153_MOESM4_ESM.docx]

**Additional file 4.** **Animals genotyped for validation of the *COL2A1*: g.32476082G>A mutation in this study.** Distribution of the g.32476082G>A genotypes in the family of Energy P (normal descendants, their dams and Energy P) in the herd where the bulldog cases were observed, further 48 females from the teaching and experimental farm Ruthe (LuFG Ruthe) and 228 animals of different farms and breeds were represented with their specifically polled status. Polled genotype was verified using strongly associated markers in the polled region according to Glatzer et al. (2013)

| Animals |  | Sex and number of animals | | Polled genotype | | | Breed | Wildtype (G/G) | Heterozygous  (G/A) |
| --- | --- | --- | --- | --- | --- | --- | --- | --- | --- |
|  |  | male | female | pp | Pp | PP |  |  |  |
| Energy P and its relatives | Progeny bulldog | 1 | 2 | 2 | 1 |  | Holstein |  | 3 |
|  | Progeny normal | 2 | 3 | 3 | 2 |  |  | 5 |  |
|  | Dams of progeny |  | 8 | 8 |  |  | Holstein | 8 |  |
|  | Sperm from Energy P | 1 |  |  | 1 |  | Holstein |  | 1 |
| LuFG Ruthe | Progeny not related with Energy P |  | 48 | 48 |  |  | Holstein | 48 |  |
| Animals from other farms and breeds |  | 119 | 43 | 57 | 94 | 11 | Holstein | 162 |  |
|  |  | 45 |  | 20 | 14 | 11 | Fleckvieh | 45 |  |
|  |  | 9 |  | 1 | 7 | 1 | Fleckvieh x Holstein | 9 |  |
|  |  | 4 |  |  | 1 | 3 | Limouson | 4 |  |
|  |  | 4 |  |  | 1 | 3 | Charolais | 4 |  |
|  |  | 3 |  |  | 3 |  | Pinzgau | 3 |  |
|  |  | 1 |  |  | 1 |  | German Yellow | 1 |  |
