# Supplementary material for: Germline mutation within COL2A1 associated with lethal chondrodysplasia in a polled Holstein family
Source: BMC Genomics. 2017 Oct 10;18:762. doi: 10.1186/s12864-017-4153-0 (PMC5633883; doi:10.1186/s12864-017-4153-0)
Supplement: Supplementary file 5 — Primer pairs used for sequencing exon 42 of COL2A1 on BTA5. Primer pairs, amplicon size (AS) in base pairs (bp) and the annealing temperature (AT) are given. (DOCX 14 kb) [file 12864_2017_4153_MOESM5_ESM.docx]

**Additional file 5. Primer pairs used for sequencing exon 42 of *COL2A1* on BTA5.** Primer pairs, amplicon size (AS) in base pairs (bp) and the annealing temperature (AT) are given.

| Primer (exon/UTR) (F=forward, R=reverse) | Sequence (5′ - 3′) | Length | AT (°C) | AS (bp) |
| --- | --- | --- | --- | --- |
| Col2A1_Ex42_F | TGGAGATGATGGTCCTTCTG | 20 |  |  |
| Col2A1_Ex42_R | AGTCTCCCTGGCCTTCTCTC | 20 | 60°C | 793 |
